# Complete Genome Sequence of *Malassezia restricta* CBS 7877, an Opportunist Pathogen Involved in Dandruff and Seborrheic Dermatitis

**DOI:** 10.1128/MRA.01543-18

**Published:** 2019-02-07

**Authors:** Stanislas C. Morand, Morgane Bertignac, Agnes Iltis, Iris C. R. M. Kolder, Walter Pirovano, Roland Jourdain, Cécile Clavaud

**Affiliations:** a L’Oréal Research and Innovation, Aulnay-sous-Bois, France; b Genostar, Montbonnot-Saint-Martin, France; c BaseClear, Leiden, The Netherlands; Broad Institute of MIT and Harvard University

## Abstract

Malassezia restricta, one of the predominant basidiomycetous yeasts present on human skin, is involved in scalp disorders. Here, we report the complete genome sequence of the lipophilic Malassezia restricta CBS 7877 strain, which will facilitate the study of the mechanisms underlying its commensal and pathogenic roles within the skin microbiome.

## ANNOUNCEMENT

Malassezia restricta, one of the most abundant Malassezia species of the human skin microbiota (1–3), is considered an opportunistic pathogen associated with skin disorders such as seborrheic dermatitis and dandruff (4–6). Due to the absence of fatty acid synthase, most Malassezia yeast growth depends on the presence of host lipids and the expression of an extensive number of lipases (7–10). However, other mechanisms are likely involved in its interactions with skin and surrounding bacteria (11, 12). Currently, public databases contain three M. restricta genomes identified at either the contig or scaffold level (whole-genome sequences [WGS] of LFCZ01, AAXK01, and LFDA01) and unassembled next-generation sequencing (NGS) reads of the M. restricta KCTC 27527 strain (8), which limits comparative genomic and *in vivo* metagenomics studies. We succeeded in completely resequencing and assembling the M. restricta genome at the chromosomal level.

Genomic DNA of M. restricta CBS 7877, a strain isolated from normal human skin (13), was purchased from the ATCC (reference MYA4611D5). Sequencing was performed at BaseClear on both the HiSeq 2500 (Illumina) and PacBio RS II (Pacific Biosciences) platforms. The Illumina library was obtained following the Nextera protocol (Illumina). The Illumina 125-base paired-end short reads (4,849,647 reads after quality control with FastQC version 0.10.0; 1.222 Mbp in total; coverage, 167×) were trimmed and *de novo* assembled using CLC Genomics Workbench version 7.5.1 (CLC bio, Denmark). The optimal *k*-mer size was automatically determined using KmerGenie version 1.6213 with default parameters (14). For PacBio, the library was prepared using the standard procedure for the PacBio RS II instrument. PacBio reads were processed and filtered using the SMRT Analysis software suite version 2.3.0, leading to 858,347 continuous long reads (1.721 Mbp in total; coverage, 235×). Illumina contigs were then aligned with the PacBio CLR reads using BLASR (15). From the alignment, the orientation, order, and distances between contigs were estimated via SSPACE-LongRead Scaffolder version 1.0 (16) and gaps filled with GapFiller version 1.10 (17). The final assembly consisted of a mitochondrial plasmid (33.6 kbp, 22 tRNA genes, 31.5% GC content) and 9 scaffolds. The longest scaffold was 1,419,096 bp. The genome size was 7.26 Mbp, with a G+C content of 56.8%. Structural annotation was performed using Augustus version 3.3 (18) with the training model organism M. restricta strain KCTC 27527 (GenBank accession numbers CP030251 to CP030259). Functional annotation was completed using a combination of BLAST-p and Blast2GO (19, 20) against Pfam (version 31) and the UniProt/Swiss-Prot (release 2017-04) databases, as included in the Prokka annotation framework version 1.12. A total of 4,096 protein-coding genes cover 86.8% of the genome.

The mitochondrial sequence is identical to the previously released M. restricta CBS 7877 mitochondrion sequence (GenBank accession number KY911093), with the exception of one repeated region. A previous karyotype study found nine chromosomes for the M. restricta CBS 7877 strain (21). Here, the nine scaffolds exhibit short tandem repeats (TKAGTG, >60 bp) considered to be telomeres, as previously reported (9). Six scaffolds show telomere repeats at both ends, suggesting that these are complete chromosomes, and these hexamer-repeated motifs were not found elsewhere in the assembly. Six and three lipase coding sequences (CDS) have been identified, harboring the Pfam PF01764 and PF03583 signatures, respectively. Among the cell wall biosynthesis-related proteins analyzed, 20 chitin-chitosan-processing genes were identified by BLAST using a Malassezia globosa protein data set (19). These activities are of main importance since the M. restricta cell wall contains a very high percentage (20%) of chitosan (22), and chitosan is reported to be required for fungal virulence and persistence in mammals (23). Further investigation on specific factors and functions (such as proteases and glycosyl hydrolases [9, 12]) will be essential to a better understanding of the physiopathology of M. restricta.

### Data availability.

The complete genome sequence of CBS 7877 is available in GenBank under the accession numbers CP033148 to CP033157. The version described in this paper is the first version. Raw data have been deposited in the SRA under the accession numbers SRX5004588 and SRX5004589 and BioProject number PRJNA474956.
